# Exploring Viral Interactions in *Clavibacter* Species: In Silico Analysis of Prophage Prevalence and Antiviral Defenses

**DOI:** 10.3390/life15020187

**Published:** 2025-01-27

**Authors:** Lucía Margarita Rubí-Rangel, Josefina León-Félix, Claudia Villicaña

**Affiliations:** 1 Centro de Investigación en Alimentación y Desarrollo A. C., Carretera a Eldorado Km 5.5, Campo El Diez, Culiacán 80110, Sinaloa, Mexico; lrubi221@estudiantes.ciad.mx (L.M.R.-R.); ljosefina@ciad.mx (J.L.-F.); 2 CONAHCYT-Centro de Investigación en Alimentación y Desarrollo A. C., Carretera a Eldorado Km 5.5, Campo El Diez, Culiacán 80110, Sinaloa, Mexico

**Keywords:** prophages, domestication, bacterial immunity, defense systems, adaptation

## Abstract

*Clavibacter* is a phytopathogenic genus that causes severe diseases in economically important crops, yet the role of prophages in its evolution, pathogenicity, and adaptation remains poorly understood. In this study, we used PHASTER, Prophage Hunter, and VirSorter2 to identify prophage-like sequences in publicly available *Clavibacter* genomes. Prophage predictions were checked by hand to make them more accurate. We identified 353 prophages, predominantly in chromosomes, with some detected phage-plasmids. Most prophages exhibited traits of advanced domestication, such as an unimodal genome length distribution, reduced numbers of integrases, and minimal transposable elements, suggesting long-term interactions with their bacterial hosts. Comparative genomic analyses uncovered high genetic diversity, with distinct prophage clusters showing species-specific and interspecies conservation patterns. Functional annotation revealed prophage-encoded genes were involved in sugar metabolism, heavy metal resistance, virulence factors, and antibiotic resistance, highlighting their contribution to host fitness and environmental adaptation. Defense system analyses revealed that, despite lacking CRISPR-Cas, *Clavibacter* genomes harbor diverse antiviral systems, including PD-Lambda-1, AbiE, and MMB_gp29_gp30, some encoded within prophages. These findings underscore the pervasive presence of prophages in *Clavibacter* and their role in shaping bacterial adaptability and evolution.

## 1. Introduction

Phytopathogenic bacteria are one of the main contributors to low agricultural productivity worldwide because they cause devastating diseases in many important crops [1]. *Clavibacter* is the most economically important Gram-positive bacterial plant pathogen, with species that cause severe diseases of maize (*C. nebraskensis*), wheat (*C. tessellarius*), tomato (*C. michiganensis*), potato (*C. sepedonicus*), bean (*C. phaseoli*), and pepper (*C. capsici*) crops [2]. Because they cause significant crop losses, some species are considered quarantine pathogens in different countries [3,4]. Despite considerable efforts to mitigate the impact of *Clavibacter*-associated diseases through traditional breeding strategies and chemical control measures, their management remains challenging, partly due to the need for a comprehensive understanding of the genetic determinants underlying their pathogenicity and environmental interactions [5,6,7].

Prophages and antiviral defenses represent two interconnected components of bacterial genomes that have received increasing attention in recent years due to their roles in shaping bacterial evolution, pathogenicity, and ecological interactions [8,9]. Prophages are viral genomes integrated within bacterial chromosomes or remaining as free phage-plasmids, which can either enhance or decrease the fitness of the bacteria, impacting their behavior, genetic variability, and ecology [10]. Some prophages provide genes encoding toxins that enhance the virulence of pathogenic bacteria, as occurs in *Acinetobacter baumannii*, *Staphylococcus aureus*, and *Escherichia coli* [11,12,13]. Similarly, recent studies have highlighted the presence of prophages in closely related *Curtobacterium* genomes, which also contribute to bacterial lifestyle, adaptation, and pathogenicity [14]. Moreover, *Bifidobacterium*, *Lactobacillus*, and *Streptococcus* have been shown to harbor numerous prophages and prophage-like components [15,16,17]. In addition to prophages, phage-plasmids, mobile genetic elements with features of both plasmids and phages, play a crucial role in bacterial fitness by facilitating horizontal gene transfer and contributing to the dissemination of virulence factors and antibiotic resistance genes [18]. Even though significant research has been conducted on prophages in relation to human bacteria, their significance in the fitness and development of phytopathogenic bacteria is comparable. For example, prophages of some of the most destructive plant pathogens, such as *Dickeya*, *Ralstonia*, *Xylella*, *Xanthomonas*, *Burkholderia*, and *Pectobacterium*, have been implicated in auxiliary genes that encode proteins that inhibit the plant immune response, degradation enzymes, and proteins involved in the secretion systems [19,20,21,22]. To make bacterial plant pathogens more competitive, prophages can encode bacteriocins that stop competitors [23], increase the resistance of bacteria to environmental stressors such as metal ions and antibacterial compounds [21,24], or boost metabolic potential to help plants survive when nutrients are limited [25].

On the other hand, bacteria have evolved a plethora of antiviral strategies to resist the invasion of viral and mobile genetic elements (MGEs), thereby influencing their susceptibility to phage predation and horizontal gene transfer [26]. These phage resistance strategies involve diverse mechanisms such as the modification of the surface receptors to prevent phage attachment [27], the action of superinfection exclusion proteins to block phage DNA injection [28], the production of enzymes that modify the bacterial genome or directly degrade phage DNA through non-specific mechanisms [29], the use of cell suicide systems to limit phage spread [30], the DNA interference by the Argonaute system to silence phage DNA [31,32], and the expression of elaborate adaptive immunity mechanisms, such as CRISPR-Cas systems, which provide targeted defense by recognizing and degrading specific phage DNA sequences, also called defense systems [33]. Among these, restriction-modification systems are particularly prevalent and crucial, as they protect bacterial genomes by cleaving foreign DNA while methylating their own DNA to prevent self-digestion [34]. In the latter, numerous defense systems with great antiphage power and extensive dispersion in bacteria and archaea have been discovered, but most of their molecular mechanisms are unknown [35,36]. Many of the antiviral systems can be carried by MGEs, such as prophages. In *P. aeruginosa*, the ϕ297 prophage modifies the structure of O-antigen subunits in lipopolysaccharides, conferring phage resistance [37]. Mycobacteriophages such as Butters, Sbash, and CarolAnn have putative toxin-antitoxin (TA) modules with membrane-associated effectors [38,39]. Some phages encode the stress alarmone guanosine pentaphosphate ((p)ppGpp) for host defense, while others encode superinfection exclusion (Sie) membrane proteins or restriction endonucleases [40,41]. The prophage-mediated antiviral systems may play an important role in limiting phage infection and increasing host competence and survival [38].

Despite the well-documented roles of antiviral defenses and prophages in various bacterial species, the distribution, diversity, and potential functions in *Clavibacter* species remain poorly understood. Given the importance of *Clavibacter*-associated diseases in agriculture and the increasing prevalence of phage-mediated control strategies for bacterial pathogens, the knowledge gap surrounding *Clavibacter* phages is striking. To date, there are available only three completely sequenced genomes of *Clavibacter* phages: CMP1 and phage 33, isolated against *C. michiganensis,* and CN1A phage isolated from *C. sepedonicus*, and the partially sequenced specific CN77 phage for *C. michiganensis* [42,43,44]. This emphasizes the necessity of addressing the impact of the viral elements in *Clavibacter* biology. In this study, we used bioinformatics and comparative genomics to identify and characterize prophage sequences within publicly available *Clavibacter* genomes, with the aim to shed light on their genomic features and functional potential. Additionally, we assessed the presence and diversity of antiviral defense mechanisms. Exploring the interplay between *Clavibacter* and their viral elements will provide valuable insights to understand the impact on pathogenicity, ecology and evolution, with potential application for creating specific strategies to reduce *Clavibacter*-related diseases and loss to agriculture.

## 2. Materials and Methods

### 2.1. Bacterial Genomes and Prophage Identification

A total of 114 genome sequences of the *Clavibacter* genus were retrieved from the GenBank database (https://www.ncbi.nlm.nih.gov/genbank/) (last accessed 31 July 2022), and listed in Table S1. Prophage identification was performed using three tools: the PHASTER (PHAge Search Tool Enhanced Release) [45], the PROPHAGE HUNTER [46], and the command line software VirSorter2 (v.2.2.4) [47]. All potential prophages were manually analyzed to depurate the raw list of prophages. For this, overlapping prophages identified on the same contig were thoroughly analyzed by sequence alignment ClustalW, and they were counted as one, adjusting the prophage length considering the non-overlapping ends. Further analysis depicting prophages type, G+C content, polylysogeny, location, and prophage length was plotted in RStudio (v.4.4.1) using ggplot2 (v.3.5.1). A comparative analysis of the host and the prophage genome sizes was conducted by correlation with the Kendall method, considering significance at a *p*-value less than or equal to 0.05.

### 2.2. Annotation of Prophage Genes

Final prophage genomes were annotated using the open-access program RAST: Rapid Annotation using Subsystem Technology [48]. Prophage genomes shorter than 4000 bp that were not accepted for RAST were annotated with GeneMarkS [49]. Then, prophage genes were evaluated to determine their similarity with bacteriophage genes based on searches with phage-limited BLASTx in the NCBI database. The analysis was restricted to tax IDs relevant to bacteriophages, namely 28883, 10699, 10474, 12333, 38018, 2100421, 186765, 10662, 10744, 79205, 10841, and 102294, using the default settings. Proteins identified as hypothetical in RAST were analyzed with BLASTx and PFAM. For searching integrases on prophage genomes, a database was constructed with phage integrase protein sequences retrieved from UniProt. Subsequently, a BLASTp analysis was performed to identify integrases in the *Clavibacter* prophages using the integrase database previously constructed. Raw results were filtered based on the following criteria: an E-value threshold of less than 0.0001, a cut-off of 50% identity, and 50% coverage and the presence of integrase-related domains with PFAM, in order to increase the probability of finding divergent integrases.

### 2.3. Functional Categorization of CDSs

Genes were classified by functional categories using the eggNOG-mapper version 2 [50] to identify the clusters of orthologous genes (COGs) of the CDSs of the prophage genes according to the eggNOG 5 database (v5). Then, a KEGG pathway enrichment analysis was performed with the advanced clusterProfiler (v4.12.6) package in R. The *p*-values were adjusted with the Benjamini–Hochberg method, and a *p*-adjusted ≤ 0.05 was set to identify the significantly enriched pathways. Subsequently, the enhanced terms were displayed through dot plots to show the metabolic pathways significantly enriched in the data.

### 2.4. Identification of Virulence Factors and Antibiotic Resistance Genes

To identify virulence genes, searches were performed using VirulenceFinder 2.0 [51] on all prophage genomic sequences. An additional survey of antibiotic resistance genes was carried out using the Comprehensive Antibiotic Resistance Database (CARD) with the Resistance Gene Identifier (RGI) option with the default parameters [52]. The detection of insertion sequences (ISs) in *Clavibacter* genomes was carried out with MobileElementFinder v1.1.2 using the default parameters [53].

### 2.5. Comparative Genomic Analyses and Defense System Detection

ANIb (Average Nucleotide Identity using BLAST) was calculated using Pyani (v0.2.12) [54] with default parameters to assess the sequence-level similarity between all prophages. The ANI similarity matrix was used to construct a heatmap using the Pheatmap function (v1.0.12) in R. The scatter plot relating prophage genome length and gene count by cluster was generated using ggplot2 (v.3.5.1) in RStudio (v.4.4.1). A hierarchical clustering of rows and columns was performed using the ward.D2/average method, while the distance between rows and columns was calculated using the Euclidean distance. All prophage cluster species levels were aligned, and amino acid synteny was visualized using Clinker [55]. The search for bacterial and phage defense systems was done using Defense Finder (https://defensefinder.mdmlab.fr/ last accessed 28 April 2024) [9].

## 3. Results

### 3.1. Identification and Prevalence of Prophages in Clavibacter Strains

**Figure 1:**
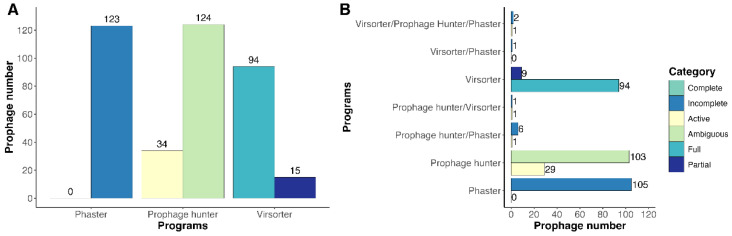
Identification of prophages in *Clavibacter* genomes using different software. (**A**) Raw identification of prophages and (**B**) identified prophages after manual curation.

To study the prevalence and diversity of prophages, genome sequences of *Clavibacter* associated with tomato (*C. michiganensis n* = 71), maize (*C. nebraskensis n* = 13), lucerne (*C. insidious n* = 7), and other crops (*n* = 24) were retrieved from the NCBI database and analyzed with three different programs. In the first instance, we identified the presence of prophages in more than 97% (*n* = 111 of 114) of *Clavibacter* genomes, identifying 123 incomplete prophages with PHASTER, 34 active and 124 ambiguous prophages with Prophage Hunter, and finally, 94 full and 15 partial prophages with Virsorter2, accounting for a total of 390 prophages (Figure 1A, Table S2). While this classification provided an initial overview of prophage diversity, further annotation revealed inconsistencies in the prediction of active prophages, where many of them were smaller than 10 kb, suggesting the majority might be inactive. We performed a manual curation because we observed that several prophages overlapped within their genomic regions and we decided to join all of them in one larger prophage. Manual curation merged several prophages into 21 prophages (highlighted in blue in Table S2), reducing the total to 353 prophages identified in *Clavibacter* genomes (Figure 1B, Table S3), an observation consistent with previous studies showing that prophages are prevalent in bacteria [56]. We noticed that only 13 out of 353 prophages were detected with two or three programs, strongly supporting their identification. Additionally, supplementary data indicate that these prophages belong to *Caudoviricetes*, a result that further validates our findings.

**Figure 2:**
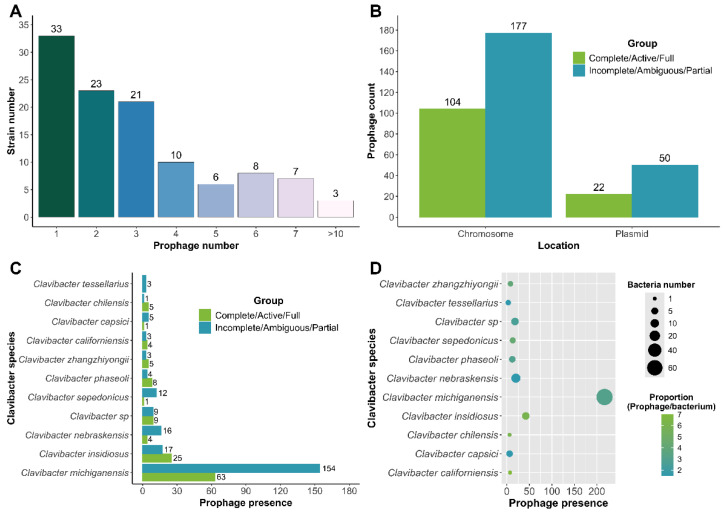
General features of prophage genomes detected in *Clavibacter* strains. (**A**) Frequency of prophages detected in each *Clavibacter* strain; (**B**) distribution of prophages according to the location in the *Clavibacter* genomes; (**C**) presence of prophages in different *Clavibacter* species; and (**D**) proportion between the number of strains and the number of prophages present per species.

Further characterization revealed that most *Clavibacter* strains harbored one prophage (32 out of 114, 28%), followed by two (24 out 114, 21%) and three (21 out 114, 18.4%) prophages, while a higher number of prophages by strain were found less frequently (Figure 2A). In fact, we observed that 17 prophages were detected in CFBP 6488 strain. Because of the diversity of prophage categories, we decided to merge prophages into two categories: Complete/Active/Full prophages, with 126 active prophages, and as Incomplete/Ambiguous/Partial prophages, with 227 cryptic prophages. Then, analysis showed that 281 prophages were located in the chromosome and 72 were phage-plasmids (Figure 2B); most of the prophages were identified in contigs, indicating that draft genomes are useful for prophage detection. Most of the identified prophages were found in *C. michiganensis,* as expected due to a higher number of analyzed genomes, and they showed a mean proportion of two phages per genome. In contrast, other *Clavibacter* species, such as *C. californiensis*, *C. chilensis*, and *C. insidious*, exhibited a higher proportion of seven, six, and six prophages per genome, respectively, although most of the analyzed genomes had fewer (Figure 2C,D). Further annotation revealed the presence of a few integrases and the absence of key phage structural and functional proteins, such as capsid or tail genes, strongly suggesting that even prophages predicted as active elements (category Complete/Active/Full) are cryptic or remanent of domesticated prophages (see below). This highlights the limitations of automatic categorization without detailed genomic analysis.

**Figure 3:**
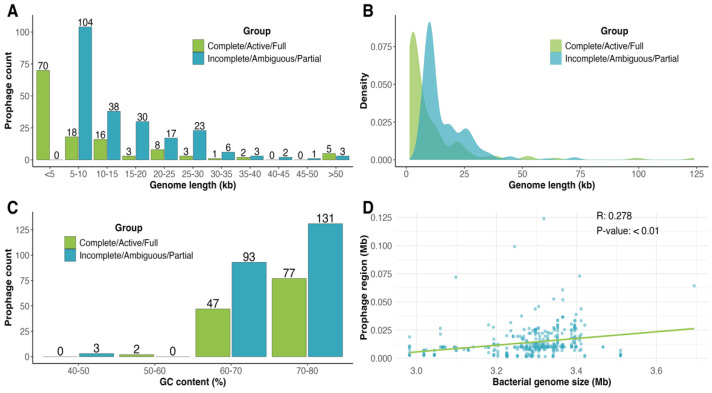
Genomic characteristics of *Clavibacter* prophages. (**A**) Distribution of prophage according to genome size (Kbp). (**B**) Density of genome length distribution. (**C**) G+C content (%) of *Clavibacter*. (**D**) Correlation between bacterial genome size and prophage genome size.

Next, the average genome size of prophages was 13.98 kbp, ranging from 1.38 to 123.96 kbp. The shortest prophage was classified as such due to the identification of a single gene with homology to a hypothetical phage gene from *Caudovirales* (formerly *Caudoviricetes*) using BLASTx limited to phage-specific genes, as described in the methodology. The genome size distribution exhibited a unimodal pattern, highly enriched with prophages up to 10 kbp in genome length (Figure 3A,B). This finding is contrary to the bimodal distribution reported by that of Bobay et al. [57], where larger prophages were interpreted as recently integrated and functional elements. In our analysis, the predominance of smaller prophages suggests that most are highly degraded. While some prophages may represent domesticated elements retained for their adaptive value, others likely reflect remnants in the process of being lost from the bacterial genome. In this context, the prophage genomes frequently accumulate mutations, and those prophage genomic regions that confer beneficial traits to the host are selected at long-term, a mechanism known as phage domestication [58]. Moreover, most of the prophages had an average G+C content of 72.16%, similar to analyzed *Clavibacter* genomes with 72.56% average GC content, although 145 (41%) prophages showed G+C contents lower than 70% (Figure 3C). Similar G+C content may suggest that most prophage-like elements are the product of long-term interactions with their *Clavibacter* hosts [59]. Linear regression analysis of host versus prophage genome sizes was very weak (R^2^ = 0.065) although it was significant (*p* < 0.001), showing that most prophages were accumulated in genomes between 3.2 and 3.4 Mb in size (Figure 3D). Taken together, these results indicated that *Clavibacter* genomes exhibit a high prevalence of cryptic prophages, many of them representing domesticated prophages.

All 126 presumptive active prophages were analyzed in public databases using BLAST limited to tax ID viruses, showing that only 26 prophages matched to known phage genomes with at least 74.6% identity, although very low coverages (lower than 9%) were observed. Matched prophages were related to *Erwinia* phage vB_EamM_Stratton, *Gordonia* phage BetterKatz, *Mycobacterium* phage ShrimpFriedEgg, *Mycobacterium* phage Kykar, and *Gordonia* phage MagicMan. Because of the low coverage, we considered all presumptive active prophages as novel.

### 3.2. Comparative Genomic Analyses of Clavibacter Prophages

**Figure 4:**
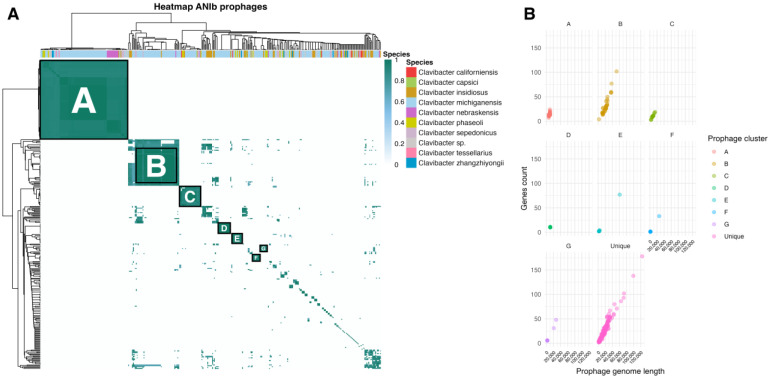
Genetic diversity and clustering of prophages in *Clavibacter*. (**A**) ANI-based heatmap. Similar prophages were classified in A to G groups. Each color in the top bar represents different *Clavibacter* species. (**B**) Relationship between prophage genome length and gene count for each *Clavibacter* prophage cluster (A–G).

To analyze the genetic diversity of prophages, we created an ANI similarity matrix and constructed a heatmap. This analysis revealed that most of the prophages were grouped into seven clusters, which shared at least of 90% identity, except for groups B and C, where some pairwise comparisons gave ANI values of at least 80% but were clustered together. The remaining prophage genomes showed identity with a few genomes or remained as single prophages, suggesting they were unique. These results indicated high genetic diversity and revealed novel *Clavibacter* prophages with unique genomes. Additionally, we analyzed the prophages’ genome length and gene content within each cluster (Figure 4B), revealing that clusters with consistent genome sizes and gene numbers, such as A, C, and D, may represent domesticated prophages, while clusters with broader variability, such as B, E, F, and G, may reflect prophages at different stages of degradation. Moreover, the analysis of unique prophages revealed a wide range of gene content and genome size. These results indicated both high genetic diversity and varying evolutionary stages among *Clavibacter* prophages, uncovering novel prophages with unique genomic characteristics.

The ANI analysis also revealed a wide range of alignment coverage values across prophages, from 0.1 to 1.0. Prophages with lower coverage values may represent elements at different stages of degradation or prophages with fewer conserved regions, while those with complete coverage likely indicate closely related prophages with minimal sequence divergence. These results suggest that coverage thresholds could influence the grouping of prophages in clusters, particularly for those in clusters C and G, which exhibited broader variability.

**Figure 5:**
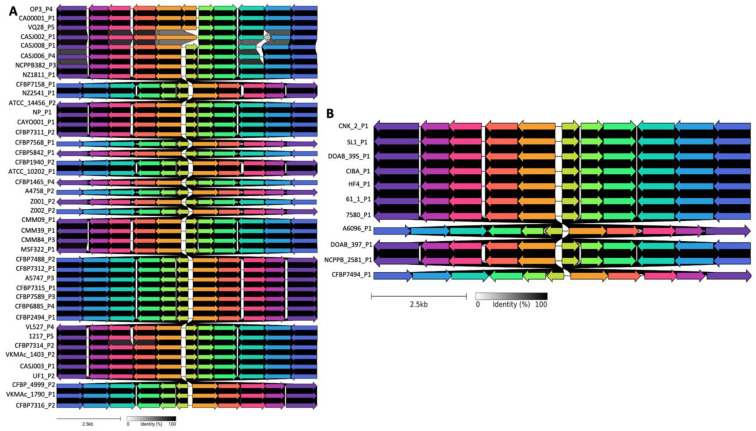
Genome alignments of clusters of *Clavibacter* prophages. (**A**) Analysis of prophages from *C. michiganensis* from cluster A. (**B**) Analysis of *C. michiganensis* prophages from group D. Shared genes across genomes are shown by arrows of the same color. The grey horizontal bar shows the degree of gene similarity between the prophage genomes.

Detailed analysis revealed that most groups of prophages were found in at least two different *Clavibacter* species, with the exception of cluster D, which was composed of only *C. michiganensis* prophages. Clusters such as A showed low variability in genome length and gene content, consistent with conserved prophages likely maintained through domestication. In contrast, clusters such as G displayed high variability in both parameters, suggesting a range of degradation states. On the other hand, cluster B showed diverse coverage values (0.1–1.0) and variability in genome length and gene content, suggesting prophages in different stages of domestication or adaptation. This pattern could reflect a transitional stage, where some prophages are more conserved, while others are undergoing diversification or degradation. The analysis of *C. michiganensis* prophages from group A showed low differences in the genomic organization, including some inversions and rearrangements (Figure 5A). A similar pattern was observed for C. *michiganensis* prophages from cluster D (Figure 5B), showing that the gene organization was conserved with some putative proteins less conserved, reflecting a lower genetic variability among prophages within the group. Strikingly, most of the prophages were classified independently of the *Clavibacter* species, which may suggest that these prophages could be acquired by the last ancestor before speciation. Alternatively, some of these prophages may represent broad host range phages; however, their degraded nature and lack of essential genes suggest they are remnants rather than fully functional elements. In the case of cluster D, only *C. michiganensis* prophages were grouped together, suggesting a species-specific group of prophages; however, we cannot rule out a potential bias caused by a reduced number of analyzed genomes representing species other than *C. michiganensis* in our study.

### 3.3. Functional Annotation of Clavibacter Prophages

**Figure 6:**
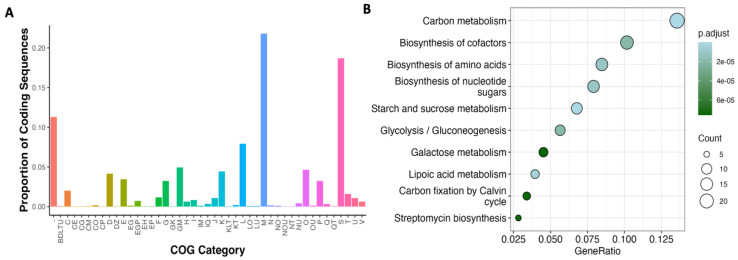
Functional categorization of prophage-encoded genes. (**A**) Distribution frequency of prophage-encoded genes classified in COGs. (**B**) Enrichment analysis of metabolic pathways using KEGG. Abbreviations: -, unclassified; B, chromatin structure and dynamics; C, energy production and conversion; D, cell cycle control, cell division, and chromosome partitioning; E, amino acid transport and metabolism; F, nucleotide transport and metabolism; G, carbohydrate transport and metabolism; H, coenzyme transport and metabolism; I, lipid transport and metabolism; J, translation, ribosomal structure and biogenesis; K, transcription; L, replication, recombination, and repair; M, cell wall/membrane/envelope biogenesis; N, cell motility; O, post-translational modification, protein turnover, and chaperones; P, inorganic ion transport and metabolism; Q, secondary metabolites biosynthesis, transport, and catabolism; S, unknown function; T, signal transduction mechanisms; U, intracellular trafficking, secretion, and vesicular transport; V, defense mechanism, Z, cytoskeleton.

In order to gain insights into the nature and function of genes, a total of 6937 CDSs (protein-coding genes) were identified in all the prophage genomes, and 3858 CDSs were retained after eggNOG analysis representing 55.6% of total CDSs. From these, 3422 CDSs were grouped in at least one of the 21 COG functional categories, while 436 out of 3858 CDSs were unclassified, representing 11.3% (Figure 6A, Table S4). Interestingly, after removing redundant descriptions of CDSs with COG, we found 594 unique CDSs descriptions, indicating that some CDSs corresponded to orthologous genes shared among the different prophages. In total, 2701 CDSs were associated with COGs with a defined function, in which the most representative categories were the M group (cell wall/membrane/envelope biogenesis) alone or in combination with other groups accounting for 26.8% (1036 out of 3858 CDSs), followed by L group (replication, recombination, and repair) alone or in combination accounting 8.2% (316 out 3858 CDSs). Interestingly, 1157 CDSs (29.9%) were hypothetical proteins (including the S group and the unclassified CDSs), which summed up to 3079 CDSs not considered by eggNOG, and represented 61% of total proteins with unknown function (4236 out 6937 CDSs). A complete annotation of prophages was done by combining the results of RAST, BLASTx, CARD, PFAM, and VFDB (Table S5).

Protein-coding genes belonging to the M group and combined categories include several proteins such as glycosyl transferases, cell wall hydrolases, and other enzymes involved in sugar metabolism. Other proteins harbor domains related to dTDP-4-dehydrorhamnose 3,5-epimerase and dTDP-glucose 4,6-dehydratase from the rhamnose pathway, likely playing an important role in the bacterial cell wall structure [60]. In addition, a total of 348 CDSs (9% of CDSs grouped with COG) are involved in carbohydrate metabolism in G, GK, GM, CG, EG, and EGP categories, which included several ABC-2 type transporters, glycosyl hydrolases, pectate lyases, and cellulases. Interestingly, we found the *celA* gene, a known virulence factor encoded by the plasmid pCM1 of *C. michiganensis* [7], was enriched in the G group. Further analysis showed *celA* was annotated as endoglucanase E1 precursors by RAST and it was present in different prophages, including the phage-plasmids LMG_3663_P4, CFIA_CsR14_P2, 1217_P3, ATCC_10253_P1_3_4, NCPPB382_P1, R1_1_P1_P4, R1_3_P1, and LMG_3663_P5_P1, and the prophages CFBP_7577_P1 and 1106_P1. Other genes related to sugar metabolism were identified, such as UDP-glucose 4-epimerase and glucose-1-phosphate thymidylyltransferase that participate in the production of precursors for the biosynthesis of complex sugars [61,62]. Several CAZymes, mainly of the glycosyltransferase type, were identified in different prophages, such as CASJ001_P1 and CIBA_P1. Furthermore, some Blastx results showed homology with hydrolase proteins from bacteriophages such as the *Erwinia* phage Fifi, suggesting a possible function in the degradation of bacterial cell wall components [63].

Based on the diversity of metabolic functions encoded by prophage genes, we performed a KEGG pathway enrichment analysis. This analysis revealed that metabolic pathways related to carbon, starch, sucrose, and galactose metabolism were enriched in prophage-encoded genes, consistent with findings observed with COG classification. Moreover, biosynthetic pathways involving cofactors, amino acids, nucleotide sugars, and streptomycin were significantly enriched. Interestingly, the streptomycin biosynthesis pathway has only been reported in *Streptomyces griseus* [64], which belongs to Actinomycetes like *Clavibacter*. We extracted the genes associated with streptomycin biosynthesis. We found four genes involved in the first metabolic steps of streptomycin biosynthesis (Figure S1), mainly as a unit in the prophage genomes. BLASTn analysis revealed that these genes showed high coverages (99%) and at least 70% identity, which suggested that these genes might be orthologous genes acquired by a phage-mediated gene transfer.

On the other hand, prophage genomes were enriched in genes that may confer advantages in adapting to diverse, challenging environments. Several genes related to copper homeostasis and resistance were identified in prophages, such as CopZ, CopG, CopD, and CopC (S group), which are involved in copper translocation. In addition, genes involved in transport and resistance to other metals were identified, such as lead, cadmium, zinc, mercury, and arsenic, including transporting P-type ATPases, chaperones, and arsenate-mycothiol transferases, which could confer an advantage to the bacteria in contaminated environments with heavy metals. Other genes encoding ABC (ATP-binding cassette) family transporters related to polysaccharide, cobalt, and Fe^3+^ transport were identified. For antibiotic resistance, we found genes encoding chloramphenicol acetyltransferases in two prophages (LMG26808_P1_3 and CASJ001_P7) using CARD analysis with perfect cut-off, which were also detected in the H group by COG analysis. Chloramphenicol acetyltransferases are known to inactivate chloramphenicol, thiamphenicol, and azidamfenicol via acetylation, being the most prevalent route for acquiring chloramphenicol resistance in bacteria [65]. Moreover, a putative helix_turn_helix multiple antibiotic resistance protein was enriched in the K group, which harbors a MarR domain (PF01047) that is characteristic of Mar proteins, suggesting a possible role in multiple antibiotic resistance [66]. Other related genes were the metallo-beta-lactamase superfamily proteins, which are often involved in the hydrolysis of β-lactam antibiotics.

Regarding virulence factors, analysis with VFDB revealed several hits associated with functions such as immune modulation, nutritional/ metabolic factors, adherence, biofilm, exotoxin, exoenzyme, accessory secretion factor, effector delivery system, stress survival, motility, invasion, and regulation. The most abundant categories were immune modulation, including glycosyltransferases, ABC transporters, capsular proteins, carbohydrate metabolism enzymes, acetyltransferases, antiporters, and some kinases; followed by nutritional/metabolic factors, the enriched genes were associated mainly with siderophores biosynthesis and transport, transporter ATPases, sugar transporters, and metal iron/manganese transporters. Then, for the effector delivery system, several proteins were identified, associated with the type VI secretion system, ATPases, transcriptional regulators, chaperones, and hydrolases. For adherence, *Clavibacter* prophages were found to encode several proteins identified as C5a peptidases, fimbria and pili biosynthesis and assembly, chaperones, metallopeptidases, and adhesins (Table S6).

**Figure 7:**
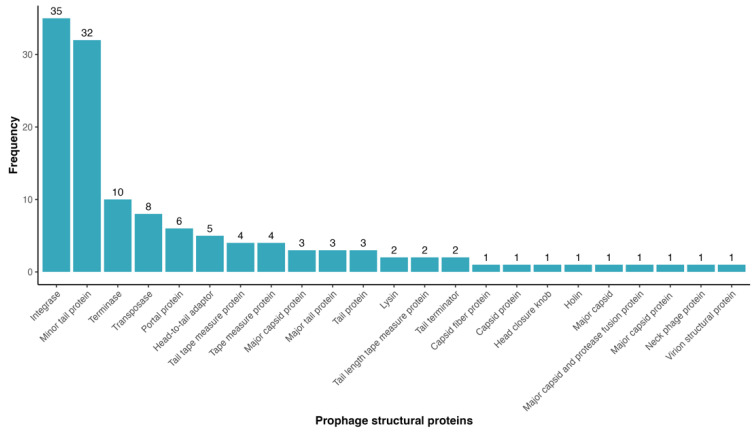
Distribution of prophage structural proteins identified in *Clavibacter* prophages. Searches were carried out using COG, BLASTx limited to viruses, RAST, and PFAM.

Based on COG analysis, we found only sixteen integrases (L group), six structural proteins (S group), and five terminases (S group). Combined annotation with RAST, BLASTx (limited to virus-related tax ids), and PFAM allowed for the identification of a total of 109 genes encoding phage-related proteins, such as integrases, major and minor capsid proteins, neck, head closure proteins, fibers, tail proteins, and head-to-tail adaptor proteins (Figure 7), which are associated with prophage integration, DNA translocation, and virion structure [67,68,69]. Despite their identification, phage-related proteins are few in comparison with all of the CDSs for predicted prophages, thus it is possible that they could be lost under the domestication events.

In addition, we found 37 transposases grouped in the L group. Previous studies have shown that transposases are highly enriched in prophages with shorter genome lengths, and the mathematical modeling including transposition events revealed that transposable elements could be relevant primarily during the initial event of domestication by inducing mutagenesis and prophage genome degradation; however, in shorter prophages representing advanced domestication events, transposable elements are lost and only beneficial genes are selected [70]. Thereby, we performed the identification of insertion elements (ISs), a kind of transposable element, finding a total of 706 ISs in 58 out 114 *Clavibacter* strains (50.87%), including 107 different ISs. From these, the most abundant were IS1121, IS1122, ISCmi2, and ISPfr17 (Table S7). However, after a detailed examination, we found that only 29 prophages contained ISs. The presence of ISs in the prophage could interfere with the activation of viral genes, influence the ability of these viruses to replicate and spread, and facilitate horizontal gene transfer [71]. ISs may be important in initial events of inactivation and domestication, but the ISs are generally lost in advanced genomic decay, similar to the observed pattern in *Clavibacter* prophages.

Taken together, we hypothesized that the underrepresentation of integrases, transposases, and terminases may indicate an advanced stage of domestication. Some studies have shown that integrases are maintained through evolution, maybe mediating the horizontal gene transfer of selfish elements, such as satellite phages, or in the process of recombination [70]; meanwhile, transposases are present in the first step of prophage inactivation but absent in advanced steps of domestication. Although Prophage Hunter and Virsorter2 algorithms detected active and complete prophages, our analysis suggests that most *Clavibacter* prophages probably are cryptic and domesticated prophages with higher levels of genomic decay.

### 3.4. Diversity of Defense Systems

**Figure 8:**
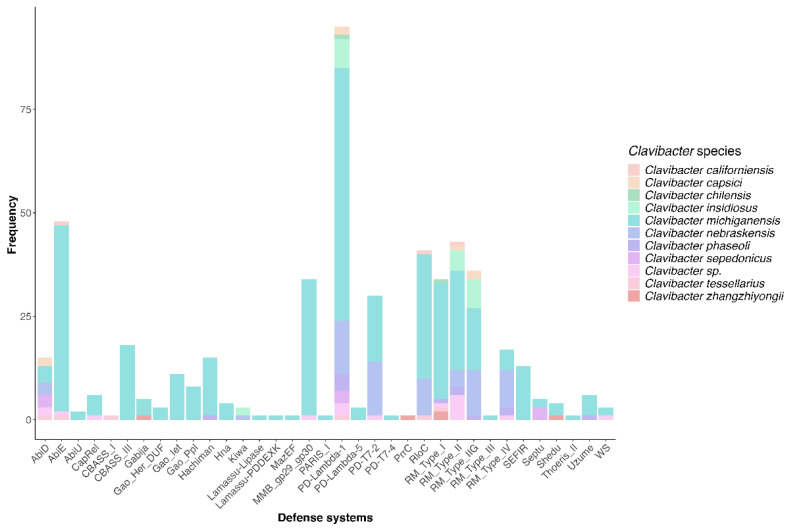
Diversity of defense systems harbored by *Clavibacter* strains. Most of the defense systems were enriched in *C. michiganensis* due to more genomes of this species being analyzed in comparison with the others.

Considering that the identified prophages seem domesticated due to long-term interaction with the host, we supposed that *Clavibacter* has been infected by prophages at a low rate, or that the few prophages reflect strong phage defense mechanisms [9]. This reasoning led us to speculate that the diversity of the immunity defense system is poor in these bacteria, since phage infection would not cause selective immunity. To explore this hypothesis, we performed a search of defense systems harbored by *Clavibacter* genomes, and strikingly, we found that 111 out of 114 genomes contained at least one defense system, accounting for a total of 505 defense systems distributed across the genomes. Moreover, these strains exhibited 35 types of immunity systems (Figure 8, Table S8). The observed diversity of defense systems suggests that *Clavibacter* may have robust mechanisms to counteract phage infections despite the low number of prophages identified. Interestingly, none of the analyzed genomes harbored the CRISPR-Cas system, indicating that this system is not found in *Clavibacter*. Meanwhile, the PD-Lambda-1 was present in 95 out 114 of *Clavibacter* genomes, followed by Abi (AbiE) in 48 genomes and restriction-modification systems (RM), MMB_gp29_gp30, and PD-T7-2 in 131, 34, and 30 genomes, respectively. In bacterial restriction-modification, the restriction endonuclease is crucial in giving tolerance against foreign DNA [72]. The MMB gp29-gp30 system is a defense mechanism identified in the MichelleMyBell (MMB) temperate mycobacteriophage, which consists of a toxic protein (gp29) and a putative membrane protein (gp30). The mechanism is unknown, but it has been determined that this protein complex helps bacterial defense against lytic phages [73]. These results suggest that *Clavibacter* exhibits a complex repertoire of defense systems despite lacking CRISPR-Cas.

Since phage predation is a potential threat, prophages may encode anti-phage defense genes to protect their host. Thereby, we searched defense-associated genes in prophages, and we found that only 12 prophages were predicted for the AbiU, RM_Type_IIG, MMB_gp29_gp30, AbiE, CapRel, PD-Lambda-1, Hachiman, and Gabija systems within their genomes. Other defense systems were less than 9000 bp upstream or downstream of four prophage genomes (Table S8). Because Hachiman and Gabija have not been reported in prophages, we performed a survey in GenBank in viral genomes, and we detected the presence of only *gajAB* genes in phage PfaC02b from *Pseudomonas*. Genomic surveys from other defense systems using BLAST revealed the presence of *darT* in *Pseudomonas* phage Zuri, *kwaAB* in phage PfAC02a, and the Thoeris anti-defense 1 protein (*ths-1*) from *Bacillus* phage Jack Rabbit. Detailed analysis with PFAM, RAST, and BLAST revealed that *Clavibacter* prophages harbored genes encoding proteins related to toxin-antitoxin (TA) systems, such as the HicA toxin and the HicB-like antitoxin from the hicAB system; the yoeB-like toxin of the YoeB/YefM systems; and a complete VapBC TA. Toxin-antitoxin was initially found as a selfish genetic element on plasmids but has been identified in bacterial chromosomes and mobile genetic elements as phages [74,75]. These results lead us to speculate that components of several defense systems, including those described only in bacteria, might be encoded by prophages.

## 4. Discussion

Despite the fact that *Clavibacter* species are considered devastating and quarantine pathogens in several important economic crops, very few studies have been conducted to explore the contribution of viral elements in their pathogenesis and evolution. To date, several studies in other relevant phytopathogens, such as *Ralstonia* and *Erwinia*, have demonstrated the presence of numerous active and inactive prophages, which contribute to genetic variability and confer diverse properties affecting bacterial adaptation to new environments and competition within microbial communities [76,77]. This study provides novel genomic insights about the diversity of prophage-like elements and defense systems across different *Clavibacter* species genomes. To improve the detection of prophages, we employed three software programs, each using unique criteria and methods to identify prophages. VirSorter2 uses a viral sequence identification system by examining signature genes, gene density, and other genomic features, accurately distinguishing viruses [47]. On the other hand, PHASTER employs searches based on sequence homology by comparison with known phage databases, having limitations in identifying unknown phages or atypical sequences [45]. Lastly, Prophage Hunter combines sequence similarity and more than 24 genetic features, such as transcriptional orientation, amino acid composition and Watson–Crick ratio, among others, using a scoring system to detect prophages, although it relies on reference data and it is less effective on highly divergent sequences [46]. Despite this, Prophage Hunter allowed for the identification of most prophages, at least in part because it uses machine learning models and detailed comparisons with viral genes, improving the detection of latent or atypical prophages compared to VirSorter and PHASTER, which might miss phages that are not similar to those in the databases. Furthermore, the database’s use of machine learning models, as seen in other tools like VirFinder, and detailed comparisons with viral genes could increase the likelihood of detecting atypical prophages, but also raises the potential for false positives. These limitations underscore the need for the manual curation and careful interpretation of automated predictions to ensure the reliability of results. Nonetheless, the results obtained from this analysis clearly showed that prophage-like elements are pervasive in *Clavibacter* genomes and encode a repertoire of proteins that may enable survival and fitness.

The detailed analysis of most *Clavibacter* prophages showed them as inactive or cryptic elements (227/353), although some prophages were classified as active or full (126/353) depending on the detection software. Nevertheless, we speculated that all prophages might be inactive based on several criteria: only a few encoded integrases, terminases, and transposases; few prophage-associated ISs were identified; and genome length exhibited a unimodal distribution enriched in short length genomes. Thus, the low presence of integrases and terminases may indicate an advanced state of domestication, where prophages are no longer active in the production of complete viral particles and depend on alternative mechanisms for their persistence within the bacterial genome. Moreover, previous studies suggested that in degraded prophages, transposable elements, such as transposases and ISs, may play an initial role in mutagenesis and genomic degradation during early domestication events but become less frequent as domestication progresses and only genes of selective benefit to the host are maintained [57]. Although we found more than 100 ISs in half of the analyzed *Clavibacter*, the fact that only 29 prophages contained IS activity for these elements, may imply they were lost in advanced domestication steps, which would be consistent with an evolutionary trend towards the degradation or domestication of these mobile elements in bacterial genomes [70].

Secondly, the unimodal genome length distribution indicates most prophage-like elements have undergone a process of domestication, where the integrated prophages experienced inactivating mutations, genome reorganization, and gene loss due to selective pressures over the long-term [57]. Finally, the diverse GC content distribution on prophage-like sequences suggested prophage groups are genetically diverse and that those with similar G+C content might be the product of long-term interactions with their *Clavibacter* hosts [59], supporting our hypothesis that most prophages are under advanced domestication. Unexpectedly, the prophage search for identification failed using BLAST, which may indicate that that *Clavibacter* prophages are novel, although bias in the identification process might be due to the scarce genomic information regarding *Clavibacter* phages in databases.

There was a notable variation in the presence and proportion of prophages among different *Clavibacter* species. Strikingly, the presence of several orthologous prophages across the different *Clavibacter* strains may suggest that they were derived from a single prophage present in the last ancestor, indicating vertical transmission [57]. Alternatively, the distribution of closely related prophages could reflect a broader host range, allowing these prophages to infect and integrate into multiple *Clavibacter* strains independently [78]. A higher number of prophages in specific genomes could be related to species-specific characteristics, such as their ability to acquire exogenous genetic material through prophages, which could influence their evolution, pathogenicity, or adaptation [12,77,79]. Moreover, the presence of phage-plasmids may offer adaptive advantages since their functions as plasmids and phages represent more pathways for the spreading of genes, improving host fitness [80]. Genomic analysis on phage-plasmids showed that their genes overlapped with plasmids and phages, indicating that phage-plasmids mediate gene exchange among prophages and plasmids, including genes associated with core functions, defense systems, and antibiotic resistance [18]. Other studies highlight the interaction and impact of phage-plasmids in phytopathogens, such as the *Ralstonia solanacearum* species complex [59]. Thereby, prophages on plasmids contribute to host bacteria’s genetic diversity and functionality.

The presence of prophage-like elements in *Clavibacter* strains indicates that they play important roles in bacterial survival, adaptation, and fitness, which is consistent with the fact that prophages harbored a repertoire of genes associated with relevant functions, such as virulence factors, antibiotic resistance, tolerance to metals, siderophores, and adhesion, among others functions. In *Streptococcus pyogenes*, the partial loss of genes in prophages maintains key genes for virulence, allowing an advantage in colonization and persistence in their host [17]. Moreover, the annotation of prophage-encoded genes revealed a remarkable frequency of genes associated with sugar and DNA metabolism, indicating an adaptation towards metabolic functions that could benefit the host, optimizing nutrient availability and facilitating adaptation to adverse environments, such as resistance to heavy metals. The presence of genes related to copper and other metal metabolism suggests that prophages may contribute to homeostasis and resistance to toxic elements, which could confer selective advantages to *Clavibacter* in agricultural environments. In *Curtobacterium*, prophages encode enzymes like glycosidases and peptidases, contributing to adaptation and offering biotechnological potential [14]. Altogether, these genes may contribute to the ability of *Clavibacter* strains to adapt to new environments [81]. On the other hand, prophages affect the host in diverse ways upon integration, such as causing mutagenesis and gene disruption, modifying levels of gene expression from the host, facilitating recombination by acting as a hot spot, or protecting from attacks of similar phages [59]. Nonetheless, some studies propose that the acquisition and loss of adaptive prophage genes are constantly occurring in bacterial genomes, favoring the flux of genetic information and their evolution and ecological adaptation [57,82].

On the other hand, the presence of diverse immunity systems suggests that *Clavibacter* is under selective pressure to keep them, indicating that the bacteria are the target of phages. Unlike other bacteria, *Clavibacter* do not possess an endogenous CRISPR-cas consistent with previous reports [83]. Instead, other defense systems such as PD-lambda systems, Abi, Hachiman, and Gabija, among others, form the repertoire of immunity systems in *Clavibacter*. Georjon et al. [84] showed that defense systems in Actinobacteria are rare systems, such as gp29-gp30 and Wadjet, and are often encoded in mobile genetic elements, which was consistent with our results. Indeed, it was shown that 2% of the defense systems identified in Actinobacteria are encoded within prophages [84], indicating that prophages play a complementary role as drivers of host immunity.

In summary, in this study, we described the occurrence and diversity of prophage-like elements in *Clavibacter* spp. We demonstrated that *Clavibacter* prophages are pervasive and most of them are cryptic and probably have high levels of domestication. However, some phage-specific genes appear to be retained without clear immediate benefit to the host, raising questions about their evolutionary role. Interestingly, many prophages were unique, unveiling the diversity of prophages infecting *Clavibacter* species. Moreover, we identified numerous genes conferring advantageous traits that could improve host fitness and adaptation, including some genes associated with defense systems. Taken together, our findings highlight that prophages are relevant to mediating phage–host interactions, broadening our understanding of *Clavibacter* biology and evolution.

## Data Availability

A part of the data is contained within the article and supplementary materials. Additional information can be provided by the authors upon request.

## Supplementary Materials

The following supporting information can be downloaded at https://www.mdpi.com/article/10.3390/life15020187/s1. Figure S1: KEGG enrichment of streptomycin biosynthesis in prophage-encoded genes; Table S1: List of *Clavibacter* strains analyzed in this study; Table S2: Raw list of identified *Clavibacter* prophages; Table S3: Curated list of identified *Clavibacter* prophages; Table S4: Results of eggNOG categories; Table S5: Prophage_annotation_Rast_blastx_CARD_PFAM_VFDB; Table S6: VDFB results; Table S7: Results of IS identification; Table S8: Results of defense system searches.
