# Supplementary figures and images for: Exploring Viral Interactions in Clavibacter Species: In Silico Analysis of Prophage Prevalence and Antiviral Defenses
Source: Life (Basel). 2025 Jan 27;15(2):187. doi: 10.3390/life15020187 (PMC11856565; doi:10.3390/life15020187)

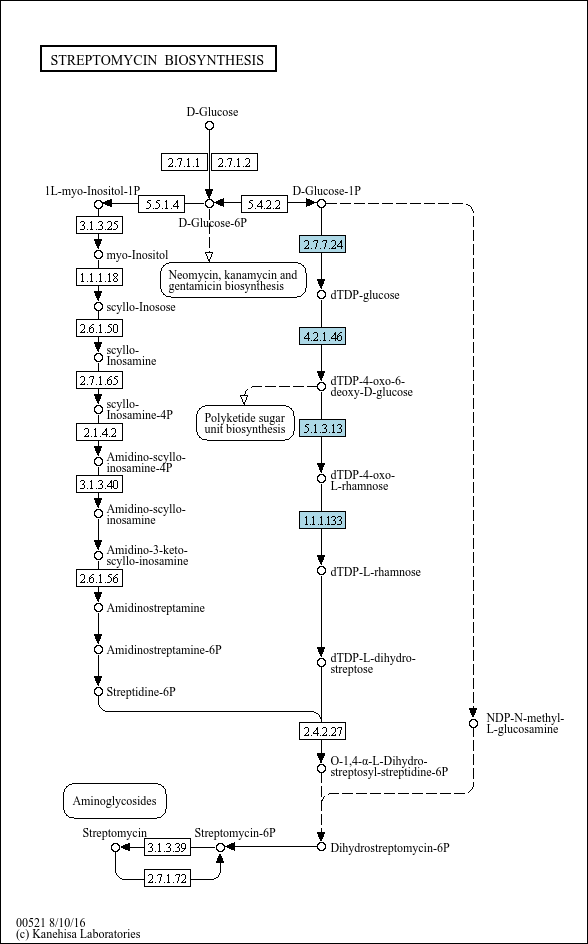

Supplement: Supplementary file 1 [file life-15-00187-s001.zip › Figure S1_KEGG enrichment of streptomycin biosynthesis in prophage-encoded genes.png]
